# Occupational noise-induced hearing loss in the mining sector of South Africa: Perspectives of occupational health practitioners on how mineworkers are trained

**DOI:** 10.4102/sajcd.v67i2.676

**Published:** 2020-03-30

**Authors:** Nomfundo F. Moroe

**Affiliations:** 1 Department of Speech-Language Pathology and Audiology, Faculty of Humanities, School of Human and Community Development, University of the Witwatersrand, Johannesburg, South Africa

**Keywords:** Awareness, Education, Hearing loss, Mineworkers, Occupational health, Health literacy

## Abstract

**Background:**

Historically, occupational health concerns associated with mining, particularly occupational noise-induced hearing loss (ONIHL), were ignored by the mining sector, policy-makers and academic researchers. As such, there is a dearth of literature related to ONIHL, especially in low- and middle-income countries such as South Africa. Consequently, mineworkers were not aware of the latent effects of excessive exposure to hazardous noise and the resultant hearing loss thereof.

**Objectives:**

The aim of this study was to explore the perspectives of occupational health practitioners (OHPs) regarding education and training of mineworkers on occupational noise-induced hearing loss (ONIHL) and its impact on mineworkers’ health.

**Method:**

Qualitative, in-depth telephonic and face-to-face interviews were conducted with 16 OHPs. Purposive and snowball sampling methods were utilised to recruit participants. Data were analysed using inductive thematic analysis.

**Results:**

Three themes emerged from the data: ‘seeing is believing’, ‘not my department!’ and ‘barriers and facilitators to raising awareness’ with two subthemes: ‘blame it on the language and level of education’ and ‘compensation pay-outs’. Superficially, OHPs believe that mineworkers are aware of the impact of noise on health; however, the OHPs are not aware on how the mineworkers are educated on ONIHL and its latent consequences. Furthermore, language, low levels of education and literacy, as well as financial constraints, are the factors found to affect education and training of the mineworkers about the risks of ONIHL.

**Conclusion:**

If the mining industry is committed in eliminating ONIHL, they should prioritise health literacy, and mines need to have an effective awareness-raising plan in place to eliminate ONIHL from every mine. This plan must consider diversity of workforce, including linguistic, as well as educational level diversity.

## Introduction

Historically, occupational health concerns associated with mining, particularly occupational noise-induced hearing loss (ONIHL), were ignored by the mining sector, policy-makers and academic researchers (National Institute for Occupational Safety and Health [NIOSH], 1996). As such, there is a dearth of literature related to ONIHL, especially in low- and middle-income countries such as South Africa. Consequently, mineworkers were not aware of the latent effects of excessive exposure to hazardous noise and the resultant hearing loss thereof (Kahan & Ross,1994).

A literature search into ONIHL in the mining sector in South Africa prior to 1994 revealed only one study by Hessel and Sluis-Cremer (1987), which was conducted on white mineworkers, with the exclusion of black workers who formed the majority of the mines’ workforce. It was only in 1994, at the apartheid-democracy twilight, that a study was conducted on black mineworkers. Kahan and Ross (1994) explored knowledge and attitudes of black mineworkers regarding ONIHL and the use of hearing protection devices (HPDs). Findings in this study revealed that firstly, mineworkers were not aware that noise was a health hazard. Secondly, their knowledge was based on personal experiences and observations, rather than on formal educational input. Thirdly, mineworkers were self-motivated to protect themselves from acquiring a hearing loss and to learn more about the effects of noise. Lastly, mineworkers complained about discomfort, feelings of insecurity because of inadequate communication and inability to hear when using HPDs. Findings of this study are consistent with Simon’s (1960) assertions that black workers were not given instructions on occupational health and safety issues as they were seen as incapable to learn.

Two decades later, in 2015, Ntlhakana, Kanji, and Khoza-Shangase explored the use of HPDs amongst mineworkers. Similar to Kahan and Ross (1994), findings revealed that mineworkers still complain about comfort, design and difficulties with work-related communication because of HPDs. Contrary to Kahan and Ross (1994), participants in this study were reportedly knowledgeable about noise exposure levels, ONIHL and appropriate use of HPDs. In the same year (2015), Edwards, Milanzi, Khoza, Letsoalo and Zungu conducted a descriptive survey to evaluate ONIHL awareness training programmes in six mines in South Africa. Their findings suggested that, firstly, there is a lack of prioritisation of commitment to awareness training by management. Secondly, a large majority of mines lacked a solid and consolidated theoretical basis for their awareness training programmes. Lastly, there were challenges with the language used during awareness training.

In 2018, Moroe and Khoza-Shangase (2018) conducted a study on the management of ONIHL in the mining sector. Findings revealed awareness training of mineworkers as one of the successes realised by the mining sector. Additionally, implementation of the revised 2014 Mine Health and Safety Council milestones on the elimination of ONIHL in the mining sector facilitated a change in the culture of the organisation as the mining industry increased focus on awareness training and education on ONIHL and its effects.

Currently, efforts on raising awareness of ONIHL may be overshadowed by the increased burden of disease, particularly human immunodeficiency virus (HIV) and acquired immune deficiency syndrome (AIDS) and tuberculosis (TB), which are the most prevalent diseases in the South African mining industry (Stuckler, Basu, McKee, & Lurie, 2011; Stuckler, Steele, Lurie, & Basu, 2013). Relying on anecdotal evidence and findings presented by Edwards et al. (2015), it appears that mineworkers are not well informed about the effects of ONIHL. Moreover, the mining industry’s efforts in targeting education of mineworkers do not seem to be successful yet. Therefore, there is a need to conduct studies to establish how mineworkers are educated on ONIHL and its consequences. This study, therefore, sought to explore the perspectives of occupational health practitioners (OHPs) responsible for implementing the training programme within the South African mining industry.

## Methods

This study is nested on a PhD study titled ‘Occupational noise-induced hearing loss in South African mines: From policy formulation to implementation and monitoring’. This article aims to explore perspectives of OHPs on awareness and training of mineworkers on ONIHL and its impact on their health.

A qualitative design, which used a naturalistic approach in seeking to understand the phenomena in context-specific settings, without the influence of the researcher, thereby eliminating the manipulation of and allowing the phenomena of interest to unfold naturally, was employed in this study (Golafshani, 2003; Patton, 2005).

Purposive snowball sampling was utilised to recruit possible participants (Valerio et al., 2016) identified from websites of companies affiliated with the South African mining industry. Participants were contacted via emails and telephonically. Furthermore, participants were requested to identify and request other appropriate participants, meeting the study inclusion criteria, on behalf of the researcher to participate in the study. Therefore, snowball sampling, as discussed by Penrod, Preston, Cain and Starks (2003), was necessary in this study because of the challenges experienced by the researcher in identifying and recruiting participants.

For inclusion in the study, participants needed to be involved in the management of ONIHL in the South African mining industry. They needed to have been employed for a minimum of 6 months to ensure that they were familiar with the hearing conservation programmes (HCPs) implemented at the mines. Ultimately, 16 participants were selected and interviewed. Participants comprised six representatives from the Mine Health and Safety Council of South Africa, seven audiologists, two ventilation and occupational health engineers and one occupational hygienist – all herein referred to as OHPs.

Data were collected through in-depth face-to-face and telephonic interviews. Telephonic interviews were conducted with participants who had time constraints and/or who were far from Johannesburg. Both telephonic and face-to-face interviews were conducted in the same manner to ensure uniformity in how data were collected. The interview structure followed the recommendations by Rubin and Babbie (2005), where the interviewer possesses a plan of inquiry as well as a set of questions. All the interviews were conducted in English and were audio-recorded for analysis. Research questions focused on the OHPs’ perspectives on awareness and training of mineworkers on ONIHL and its effects.

Reflexivity and bracketing were applied during the transcription and analysis of data as well as during the report writing process. This was carried out to guard against any bias from the researcher. After transcribing the interviews, the researcher conducted member or participant checks to ‘learn from the interviewee how well the researcher’s interpretations reflect the interviewee’s meaning’ (Morrow, 2005, p. 17).

Inductive thematic analysis was used to code the data that allowed the themes to emerge from the data (Braun & Clark, 2006). Data were analysed using six steps recommended by Braun and Clark (2006), which include familiarisation with the data, generation of initial codes, searching for themes, reviewing themes, defining themes and writing up. Representative verbatim quotations were used in the write-up of the study to provide examples of the themes that emerged from the data.

### Ethical considerations

Ethical clearance was obtained from the university’s Human Research Ethics Committee (Medical) (Protocol Number: M160264).

## Results and discussion

The following three themes emerged from the data, which are discussed in the subsequent sections: ‘seeing is believing’, ‘not my department!’, and ‘barriers and facilitators to raising awareness’ with two subthemes: ‘blame it on the language and level of education’ and ‘compensation pay-outs’.

### Theme 1: Seeing is believing

Participants were asked to share their perspectives on whether mineworkers are aware of the impact of ONIHL and its latent consequences on their hearing. The majority indicated that the workers are superficially aware of the impact of noise.

Participant 11 felt that, although the miners are aware of the impact, they only fully appreciate the impact when they develop a hearing loss:

‘I think so. Yes. But I don’t think the fact that it is permanent (hearing loss), cannot be cured. I don’t think that people realize that. I think at the back of their minds they think when they go away from the mines, back home, their hearing will get better again. I think they have this perception that while I’m in the noise, it’s damaged but once I go home and I’m not working in the mines anymore my hearing will improve.’ (P11, female, no date)

Participant 1 believes that as hearing is insidious and is an abstract concept to grasp, some workers do not actually understand the implications of acquiring a hearing loss:

‘But I think what we need to remember is that the ear is a very abstract thing for them (workers). When we tell people there is a hole in your eardrum, it’s like what are you talking about! They don’t see it. That’s the problem. Our workers are people who want to see something. You know, when I break my arm and the bone is sticking out, I can see it you know. So next time, I’m gonna [going to] (sic) be careful doing that job because my colleague was injured and I saw the blood. I think hearing loss, because it is an invisible thing to them, I think it is a difficult thing to comprehend….’ (P1, female, no date)

From the results presented above, OHPs believe that mineworkers have limited and superficial knowledge regarding noise exposure and its effects on them. The perception is that mineworkers do not fully grasp that ONIHL is irreversible. According to Kurmis and Apps (2007), workers may not be aware of the harmful nature of excessive noise exposure because of its latency and painless nature.

Mostaghaci et al. (2013) and Tye-Murray (2009) argue that ONIHL is irreversible and permanent and that once acquired, there are no guaranteed benefits from rehabilitation. Therefore, it is important that workers are made aware of this lasting negative impact of hearing loss on their quality of life. As shown in this study, although workers may be informed about ONIHL, it was observed that the medical information presented at the time of diagnosis could be misunderstood or easily forgotten (Kessels, 2003; Reese & Hnath-Chisolm, 2005), which may be the case with the mineworkers. Written or spoken information presented at induction is essential; however, it may not be sufficient to empower mineworkers in such a way that they actively practice good occupational health. The fact that hearing loss is an abstract concept calls for reinforcing of health literacy in the mining sector. Health literacy is ‘the degree to which individuals have the capacity to obtain, process, and understand basic health information and services needed to make appropriate health decisions’ (National Academies of Sciences and Medicine, 2016).

Education and motivation are a priority in minimising hearing loss in the mines as they create opportunities for both management and employees to discuss and agree on commitments, communication lines and cooperation (Byrne, 2005). Training serves to explain various and pertinent topics, such as the effects of noise on hearing and the purpose and value of wearing HPDs. During training, advantages and disadvantages of the hearing protectors being offered could be discussed in detail, with an opportunity for highlighting miners’ and operators’ responsibilities in maintaining noise controls (Byrne, 2005). If individuals understand the reasons and the benefits of an HCP, they are more likely to participate, especially if training addresses the specific needs of individuals exposed to excessive noise. Furthermore, training may be more effective when conducted continually and around the annual hearing tests and in small groups (Byrne, 2005). There is, therefore, a need to aggressively promote education within the mining sector in order to combat ONIHL.

### Theme 2: Not my department!

Participants were asked to elaborate on how workers are educated on the impact of ONIHL on their hearing. Responses highlighted that participants are not knowledgeable on how workers are trained. It should be noted that these participants are OHPs and they should either be involved in the education or be informed on whose role it is to inform workers regarding the effects of ONIHL:

‘I don’t know how it’s done. It’s in the law that the worker must be trained, how to clean and use his protection but it’s also done at the Safety department. Also nothing to do with the Medical department (sic). So it’s definitely in the law but I don’t know which programmes or how many hours or you know, exactly how it’s done.’ (P11, female, no date)

Participant 7 stated the following:

‘I don’t know. I’m not 100% sure. I’m sure that they get information on that. For example, at XXX mine there was a big… like a waiting area. And there is a TV that shows all these videos and information. I know there, they have something. But I’m not 100% sure.’ (P7, female, no date)

Participant 1 admitted the following:

‘To be honest, we haven’t done a campaign solely on noise and I think this year, depending on funding we will look into such a campaign.’ (P1, female, no date)

It is of concern that OHPs are not informed regarding mineworkers’ education and training on ONIHL; however, they claim that mineworkers are aware of the effects of noise on their health. Byrne (2005) asserts that before implementing HCPs, mines should address administrative issues, where company regulations as well as individuals’ responsibilities and roles are identified and enforced. From the responses above, it is not clear that OHPs are not clear about who is responsible for educating mineworkers on ONIHL. In South Africa, evidence indicates that there are a few audiologists employed in the mining sector as mines prefer services of audiometrists who are considered more cost-effective (Moroe & Khoza-Shangase, 2018). Edwards et al. (2015) commented that mines have a lack of qualified professionals who possess knowledge and skills on how to teach adults to achieve health promotion and behavioural change. Therefore, these findings highlight the important role of audiologists in actively participating in educating and training mineworkers regarding the impact of ONIHL on their health.

In the United States, audiologists may firstly provide programmes tailored to the needs of the audience such as HCP team members, employee work groups, supervisors and upper management. Secondly, they may develop or recommend appropriate educational materials. Thirdly, they may instruct in-house staff in effective methods of motivating and educating workers. Fourthly, audiologists contribute to management education by preparing articles for publication or speaking before trade and management groups. Lastly, they maintain up-to-date knowledge of pertinent local, state and federal regulations in order to provide management with accurate information concerning these matters (American Speech-Language-Hearing Association, 2004). Hence, there is a need in the South African mining industry for audiologists to play an active role in providing direction on the material and strategies in teaching mineworkers and management on ONIHL and its effects on individuals exposed to excessive noise.

### Theme 3: Barriers and facilitators to raising awareness

Two subthemes were identified regarding the theme: ‘barriers and facilitators to raising awareness’. The first subtheme (‘blame it on the language and level of education’) was on the part of OHPs, whilst the second subtheme (‘compensation pay-outs’) was in relation to mineworkers. These barriers were also cited as facilitators, in that, if they are overcome, they can enable a conducive environment and positively contribute towards promoting awareness and education amongst mineworkers.

#### Subtheme 3.1: Blame it on the language and level of education

Participants were asked to share possible barriers to teaching mineworkers about ONIHL and its effects. Language and levels of education were highlighted as the biggest barriers.

Participant 8 admitted that language differences contribute to the difficulty in teaching:

‘Obviously, I know that I’m lacking in my communication with my patients. I always feel that it is important to have good communication with all my patients that I see. Most of them can’t even speak English or Afrikaans. So that is a struggle for me. I really try my best.’ (P8, female, no date)

Participant 5 shared a similar experience:

‘They do understand the basics because they have been getting screened for a very long time. But getting to the details… that is a struggle for me because I’m not able to communicate with them in a language that they understand.’ (P5, female, no date)

Participant 14 mentioned the levels of education as barrier as well:

‘It’s difficult; you know your skills levels, your education levels generally in the mining industry is not that high for your average worker. Most of them do not have proper education and you know, so I think it’s a very difficult thing.’ (P14, female, no date)

Nelson Mandela, the late former president of South Africa accurately captures the importance of language when engaging with people: ‘If you talk to a man in a language he understands, that goes to his head. If you talk to him in his language that goes to his heart’ (Laka, 2014, n.p.). This quote rings true in this context where language seems to be a barrier in achieving the desired results in the training of workers. A study conducted by Edward et al (2015) indicated that 30% of the training on ONIHL in the mines was conducted in English only, 30% in Zulu and English and the remaining 40% in the employee’s language of choice. This is not an ideal situation if one considers the racial and linguistic profile of mineworkers in South African mines, which mainly comprised of black African language speakers. Moreover, South Africa is a multilingual country with 12 official languages. Using English as a chosen mode of communication still excludes workers who may not be proficient in the English or Zulu languages. The 2011 census indicated that isiZulu is the most spoken language with English ranking at number four (South African Statistics, 2016). This raises a challenge in the training of workers on the dangers of noise. Furthermore, as Africa is a developing continent and South Africa, because of its infrastructure, continues to be viewed as the richest developing country in Africa, this has led to people from neighbouring countries migrating to South Africa to seek better job opportunities in the mining industry (Van Averbeke, 2003). This has resulted in a culturally and linguistically rich and diverse environment. Occupational health practitioners, therefore, need to take language and culture into account when planning worker-training initiatives within the mining sector.

In addition to the language barrier, challenges with literacy levels of mineworkers were also reported as a barrier in this study. The report of literacy levels being a barrier is contradictory to the report that literacy levels have improved amongst mineworkers in the past few years, especially with the advent of millennials in the mining sector (Smit & Mji, 2012). Edwards et al. (2015) indicated that the content of training materials used to train workers was the same as that used for managers and other levels of workers. These findings shed light on some of the reasons that mineworkers are reported to have superficial knowledge on ONIHL and its impact.

Therefore, to overcome the literacy and language barriers, information should be matched to the worker’s levels of understanding by utilising a range of modalities such as verbal, visual and printed material to enhance learning and improve awareness whilst observing literacy, cultural and linguistic relevance. In this way, even complex concepts could be understood if appropriate communication skills are applied (Zuhlke & Engel, 2013). It is known that physicians’ explanations and the level of patients’ understanding significantly affect treatment adherence, treatment outcome and patients’ satisfaction (Clayton, 2010). The same argument applies to OHPs. If trainers explain concepts at the level of mineworker’s understanding, workers could have deeper knowledge and understanding of the consequences of excessive exposure to noise at the workplace, and they are more likely to adopt and practice preventive change in behaviour at the workplace.

#### Subtheme 3.2: Compensation pay-outs

Participants were asked why mineworkers still present with a high incidence of ONIHL if they are aware of ONIHL and its effects. Responses indicated that socioeconomic factors may be at play:

‘I definitely think that compensation plays a very big role. I definitely think that it plays a very big role because unfortunately we see a lot of people who pretend to have a hearing loss because they want compensation. It is something that in a very sad way motivates them not to look after their ears because they think they gonna (sic) get money.’ (P7)

Participant 6 confirmed financial gain as a major contributor:

‘…You know because there are production bonuses in the industry. Sometimes people feel… people then sacrifice Health and Safety because they are chasing production bonuses.’ (P6)

Participant 2 also shared her concern about the observed practice:

‘…[*W*]hen we intervene, then the exposed individual says ‘I can hear’ because there are incentives for high production and you know so and so is strong. That same individual will go back to drilling because he knows that his team depends on him. So there are those dynamics.’ (P2)

Participant 2 further elaborated:

‘So then the discussion shifted to should there not be indicators of health and safety that are included in the bonus. So… you know, sort of be remunerated or rewarded for high production but also at the same time keeping or maintaining health and safety.’ (P2)

Workers may be aware of ONIHL and its effects; nevertheless, socioeconomic difficulties may play a role in health practices of mineworkers. South Africa as a whole is faced with high levels of unemployment, high burden of disease as well as increased cost of living. Historically, mineworkers were unskilled and uneducated and were refused skill training to improve their skills and salaries (Kane-Berman, 2017). The potential of a financial incentive for compensation for loss of hearing is understandably a possible way of addressing their financial needs and therefore not taking heed of any training that they may have received or knowledge of the consequences that a mineworker may have regarding excessive exposure to hazardous noise levels. Although current findings are based on OHPs’ speculations, they, nevertheless, highlight the plight faced by workers in meeting their financial obligations. It is of concern that some miners may resort to exposing themselves to excessive noise for financial gains. However, it is also positive that the mining industry is aware of this predicament; hence, the attempt is to balance high production with maintaining health and safety as alluded to by Participant 2.

## Conclusions

One of the most important components of an effective HCP is the education and motivation of workers and management (American Speech-Language-Hearing Association, 2004). Success of programmes relies on the involvement of all stakeholders to ensure that objectives and outcomes are identified and achievable (Leonard, 2010). Additionally, stakeholders are likely to support initiatives if they are involved in the decision-making process (Consultative Forum on Mining and the Environment, 2002), as without their support, initiatives may be ignored, criticised, resisted or even sabotaged (US Department of Health and Human Services, 2011). Mineworkers are primary stakeholders in the management of ONIHL, and if the mining industry is committed to eliminating ONIHL, they should involve mineworkers in decision-making processes to ensure active participation, empowerment and support for the programme. Furthermore, audiologists also need to take an active role in educating and training mineworkers and other professionals on the impact of excessive noise at the workplace. Current findings are important as they raise issues that audiologists must take into consideration for their role in HCPs as far as awareness training is concerned. However, these findings should be interpreted with careful consideration of the identified limitations of the methodology. The study is based on the perspectives of OHPs; therefore, these findings must be interpreted with caution as they may be different from the perspectives and lived experiences of mineworkers.
